# Efficacy of 5-hydroxytryptamine 3 receptor antagonists versus metoclopramide for preventing nausea and vomiting during azacitidine chemotherapy in patients with myelodysplastic syndromes or acute leukemia: a retrospective observational study

**DOI:** 10.1186/s40780-025-00444-3

**Published:** 2025-04-24

**Authors:** Yoshinori Wakasugi, Yoshito Ikeda, Satoshi Noda, Makoto Murata, Shin-ya Morita

**Affiliations:** 1 Department of Pharmacy, Shiga University of Medical Science Hospital, Otsu, 520-2192 Shiga Japan; 2 Department of Pharmacotherapeutics, Shiga University of Medical Science, Otsu, 520-2192 Shiga Japan; 3 College of Pharmaceutical Sciences, Ritsumeikan University, Kusatsu, 525-8577 Shiga Japan; 4 Department of Hematology, Shiga University of Medical Science, Otsu, 520-2192 Shiga Japan

**Keywords:** Ramosetron, Granisetron, Metoclopramide, Azacitidine, Nausea, Vomiting, Antiemesis

## Abstract

**Background:**

5-Hydroxytryptamine 3 receptor antagonists (5-HT_3_RAs) and dexamethasone are recommended to prevent azacitidine-induced nausea and vomiting. In clinical practice, 5-HT_3_RAs or metoclopramide is often used without dexamethasone. In this study, we aimed to determine whether 5-HT_3_RAs or metoclopramide is more effective for suppressing nausea and vomiting during azacitidine-based chemotherapy.

**Methods:**

This study was a single-center retrospective observational study. Patients with myeloid malignancies receiving azacitidine-based regimens were treated with a 5-HT_3_RA (ramosetron or granisetron, *n* = 32) or metoclopramide (*n* = 18) for preventing nausea and vomiting. The occurrence of nausea and vomiting was assessed using total control (TC), complete control (CC), and complete response (CR) rates (chi-squared test), and the time to the first emetic episode or rescue medication (Cox proportional hazard regression analysis).

**Results:**

The 5-HT_3_RA group had significantly higher rates of TC, CC, and CR than the metoclopramide group (84% vs. 22%, 91% vs. 33%, and 91% vs. 33%, respectively). The time until the first emetic episode or rescue medication was also significantly longer in the 5-HT_3_RA group than in the metoclopramide group (*p* < 0.001).

**Conclusions:**

5-HT_3_RAs may prevent azacitidine-induced nausea and vomiting more effectively than metoclopramide.

**Trial registration:**

Retrospectively registered.

## Introduction

Chemotherapy-induced nausea and vomiting (CINV) is a severe adverse event associated with cancer therapy, which impairs patients’ quality of life and reduces their treatment compliance [1,2]. The frequency of CINV is strongly dependent on the emetogenicity of the anticancer agents. Based on the percentage of nausea and vomiting within 24 h after administration of various anticancer agents without antiemetic prophylaxis, four categories of emetic risk are defined (severe > 90%, moderate 30–90%, mild 10–30%, minimum < 10%).

Ramosetron and granisetron, 5-hydroxytryptamine 3 receptor antagonists (5-HT_3_RA), suppress CINV. On the other hand, metoclopramide possesses an antiemetic activity through dopamine D_2_ receptor antagonism in the chemoreceptor trigger zone. Azacitidine is an anticancer agent administered for treatment of myelodysplastic syndrome and acute myeloid leukemia. Azacitidine is classified as a moderate emetic risk drug by the Japan Society of Clinical Oncology (JSCO) Clinical Practice Guidelines for Antiemesis [3], and 5-HT_3_RAs and dexamethasone are recommended for prophylactic use concomitantly with azacitidine [3]. However, in clinical practice, 5-HT_3_RAs or metoclopramide are often used without dexamethasone to avoid the risk of infection induced by the immunosuppressive action of dexamethasone. Side effects with small doses of metoclopramide are less frequent [4]. In the phase II clinical trial of 5-HT_3_RAs conducted in Japan, although 34.0% of patients experience nausea, grade 4 nausea is not observed during azacitidine treatment [5]. However, there are no comparative studies between 5-HT_3_RAs and metoclopramide for preventing emesis, and thus, it is still unclear whether 5-HT_3_RAs or metoclopramide is more effective for suppression of CINV. Therefore, the aim of the current study was to retrospectively compare the antiemetic effects of 5-HT_3_RAs and metoclopramide during chemotherapy with azacitidine.

## Methods

### Patients

This was a single-center retrospective observational study. In the present study, eligible patients with myeloid malignancies (myelodysplastic syndromes or acute myeloid leukemia) aged between 20 and 95 years received azacitidine or azacitidine plus venetoclax regimen as initial chemotherapy during hospitalization and were treated with either a 5-HT_3_RA (ramosetron or granisetron) or metoclopramide as prophylactic therapy for preventing emesis at Shiga University of Medical Science Hospital (July 2010 to March 2023). Patients with any of the following conditions were not included in the study: (i) concomitant use of any drugs with antiemetic activity (e.g. 5-HT_3_RAs other than ramosetron or granisetron, dopamine D_2_ receptor antagonists other than metoclopramide, neurokinin-1(NK1) receptor antagonists, corticosteroids, benzodiazepine and antihistamines), (ii) symptomatic brain metastasis, (iii) hypercalcemia, and (iv) gastrointestinal obstruction.

### Administration of drugs

In the azacitidine regimen, azacitidine (75 mg/m^2^) was administered from Day 1 to Day 7. In the azacitidine plus venetoclax regimen, azacitidine (75 mg/m^2^ on Day 1–7) and venetoclax (100 mg/day on Day 1, 200 mg/day on Day 2, and 400 mg/day on Day 3–28) were administered after meals. Immediately prior to the azacitidine administration, patients received either a 5-HT_3_RA (0.1 mg of ramosetron or 3 mg of granisetron) (5-HT_3_RA group) or 5 mg of metoclopramide (metoclopramide group) as prophylactic therapy for preventing emesis.

### Assessment of nausea and vomiting

The occurrence of nausea, vomiting, or use of rescue medication during 0–288 h from initiation of azacitidine were assessed. Nausea and vomiting were monitored twice a day (morning and evening) during 0–288 h from initiation of azacitidine. The primary efficacy end point was the total control (TC) rate, which was defined as the proportion of patients with no vomiting, no nausea, and no use of rescue medication during 0–288 h from the initiation of azacitidine. The secondary end points were the complete control (CC) rate, defined as the proportion of patients with no vomiting, no or mild nausea (Grade 0–1, the Common Terminology Criteria for Adverse Events v5.0), and no use of rescue medication, and the complete response (CR) rate, defined as the proportion of patients with no vomiting and no use of rescue medication during 0–288 h from the initiation of azacitidine. According to the Common Terminology Criteria for Adverse Events, grade 1 nausea is defined as loss of appetite without alteration in eating habits, grade 2 nausea is defined as decreased oral intake without significant weight loss, dehydration or malnutrition, and grade 3 nausea is defined as inadequate oral caloric or fluid intake; tube feeding, total parenteral nutrition, or hospitalization indicated. The severity of nausea, including anorexia, was retrospectively determined by the investigators based on the documentation in the electronic medical records by nurses. Other secondary end points included the time to the first emetic episode (vomiting or nausea) or the first use of rescue medication up to 288 h from the initiation of azacitidine.

### Statistical analysis

Unpaired *t*-test, chi-squared test, and fisher’s exact test were used to examine differences in characteristics between 5-HT_3_RA and metoclopramide groups. Chi-squared test was used to compare TC, CC, and CR rates between the two groups. The intergroup differences in the time to the first emetic episode (vomiting or nausea) or the first use of rescue medication were analyzed using Cox proportional hazard regression analysis. Statistical analysis was performed using SPSS software (IBM, Armonk, NY, USA).

## Results and discussion

### Patient baseline clinical characteristics

In this study, we identified a total of 85 patients but excluded 35 patients because of concomitant use of drugs with antiemetic activity (e.g., aprepitant, palonosetron, clonazepam, olanzapine, or corticosteroids) other than 5-HT_3_RAs (ramosetron and granisetron) and metoclopramide. The patients’ baseline clinical characteristics are presented in Table 1. Patients in the 5-HT_3_RA group were administered either ramosetron (*n* = 27) or granisetron (*n* = 5). There was no significant difference in age, sex, regimen, types of myeloid malignancies, route of administration, or relative dose intensity of azacitidine and venetoclax between 5-HT_3_RA and metoclopramide groups.

**Table 1:** Patient characteristics

	5-HT_3_RA (*n* = 32)	Metoclopramide (*n* = 18)	*p* value
Age (years), median (range)	67.0 (34–88)	63.5 (18–78)	0.357^a^
Sex (male), number (%)	21 (65.6)	11 (61.1)	0.750^b^
Regimen, number (%)			0.642^c^
Azacitidine	28 (87.5)	17 (94.4)	
Azacitidine + venetoclax	4 (12.5)	1 (5.6)	
Myeloid malignancies, number (%)			0.694^c^
MDS	26 (81.3)	16 (88.9)	
AML	6 (18.7)	2 (11.1)	
Route of administration, number (%)			0.399^c^
Subcutaneous	27 (84.4)	17 (94.4)	
Intravenous	5 (15.6)	1 (5.5)	
RDI of azacitidine and venetoclax (%)	98.9 (80.1-109.7)	97.1 (83.3-114.4)	0.414^a^

Data are expressed as the median (range) or the number of patients (percentage in each group). Statistical difference was assessed by ^a^unpaired *t*-test, ^b^chi-squared test, and ^c^fisher’s exact test. 5-HT_3_RA, *5*-HT_3_ receptor antagonist; AML, acute myeloid leukemia; MDS, myelodysplastic syndromes. Relative dose intensity (RDI) (%) = dose intensity (mg/m^2^/week)/planned dose intensity (mg/m^2^/week) × 100

### Antiemetic effects

**Fig. 1:**
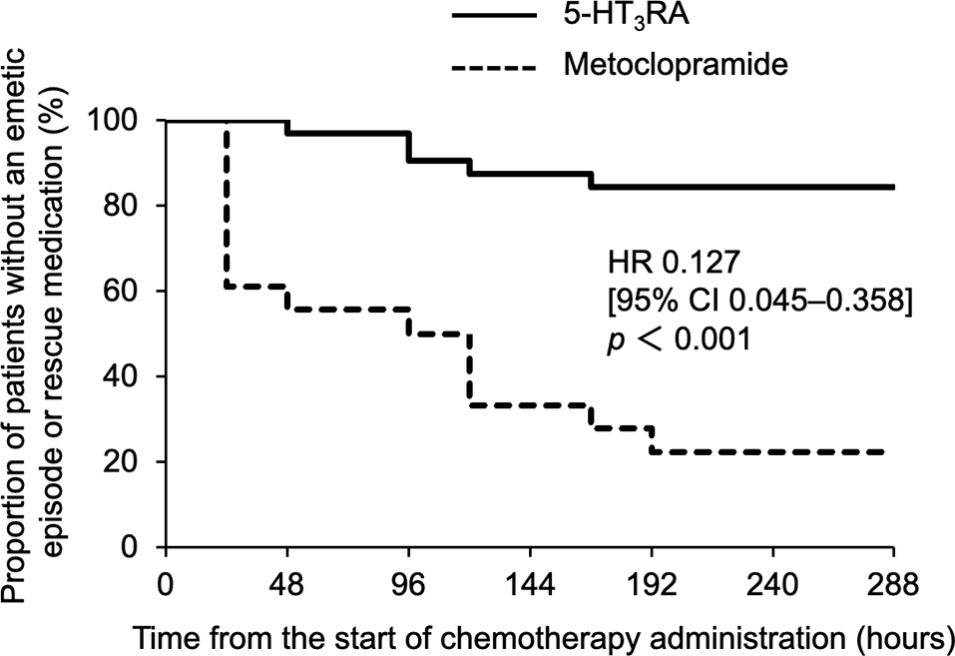
Kaplan–Meier curves for the time to first emetic episode (vomiting or nausea) and first use of rescue medication during the first 288 h following administration of chemotherapeutic drugs. 5-HT_3_RA [ramosetron (0.1 mg/body, orally) or granisetron (3 mg/body, intravenously)] (solid line), or metoclopramide (5 mg/body, orally) (dashed line) was administered once per day from Day 1 (before initiation of azacitidine) to Day 7 (HR for 5-HT_3_RA vs. metoclopramide, 0.127; 95% CI, 0.045–0.358; *p* < 0.001, Cox proportional hazard regression analysis). 5-HT_3_RA, 5-HT_3_ receptor antagonist

The proportion of patients achieving TC, CC, and CR are presented in Table 2. The proportion of patients achieving TC was significantly higher in the 5-HT_3_RA group than in the metoclopramide group (*p* < 0.001). Additionally, the proportion of patients achieving CC and CR was higher in the 5-HT_3_RA group than in the metoclopramide group (both *p* < 0.001). As shown in Fig. 1, the time to first emetic episode (vomiting or nausea) and first use of rescue medication was significantly longer in the 5-HT_3_RA group than in the metoclopramide group (hazard ratio [HR], 0.127; 95% CI, 0.045–0.358; *p* < 0.001).

**Table 2:** TC, CC, and CR rates 0–288 h after chemotherapy

	5-HT_3_RA (*n* = 32)	Metoclopramide (*n* = 18)	*p* value
TC	27 (84.3%)	4 (22.2%)	< 0.001
CC	29 (90.6%)	6 (33.3%)	< 0.001
CR	29 (90.6%)	6 (33.3%)	< 0.001

5-HT_3_RA [ramosetron (0.1 mg/body, orally) or granisetron (3 mg/body, intravenously)], or metoclopramide (5 mg/body, orally) was administered once per day from Day 1 (before initiation of azacitidine) to Day 7. TC was defined as no vomiting, no use of antiemetic rescue medication and no nausea. CC was defined as no vomiting, no use of antiemetic rescue medication and only grade 0–1 nausea. CR was defined as no vomiting, and no use of antiemetic rescue medication. Statistical difference was assessed by chi-squared test. 5-HT_3_RA, 5-HT_3_ receptor antagonist; CC, complete control; CR, complete response; TC, total control

Azacitidine is used as standard therapy for myelodysplastic syndromes [6,7]. Azacitidine plus venetoclax treatment has been reported to significantly prolong overall survival compared with azacitidine treatment in patients with untreated acute myeloid leukemia without receiving intensive remission induction therapy [8]. The National Comprehensive Cancer Network (NCCN) guidelines classify the emetic risk of azacitidine as mild and recommend the use of dexamethasone (8–12 mg), a 5-HT_3_RA [dolasetron (100 mg), granisetron (1–2 mg), or ondansetron (8–16 mg)], metoclopramide (10–20 mg), or prochlorperazine (10 mg) prior to the administration of azacitidine [9]. On the other hand, JSCO Clinical Practice guidelines classify emetic risk of azacitidine as moderate and recommend the use of a 5-HT_3_RA and dexamethasone [3]. However, there is no consensus on the optimal therapy for preventing azacitidine-induced emesis. In this study, the choice of antiemetic therapy was determined by physicians and different between patients.

NK1 receptor antagonists, 5-HT_3_RAs, corticosteroids, and olanzapine are treatment options for chemotherapy-induced nausea and vomiting, and additionally, metoclopramide and benzodiazepines are be used. Supportive care for chemotherapy-induced nausea and vomiting in hematopoietic tumors tends not to be subjected to the guidelines, and dexamethasone is rarely used, which has been reported in the JSCO survey of guideline compliance rates [10]. This is mainly due to the prevention of excess use of steroids in addition to steroids used as antitumor agents in the regimens for malignant lymphomas and multiple myeloma. Regimens for acute myeloid leukemia and myelodysplastic syndromes do not include steroids as antitumor agents, which differs from those for malignant lymphoma and multiple myeloma. Patients with acute myeloid leukemia and myelodysplastic syndromes are highly susceptible to infection, and corticosteroids, such as dexamethasone, increase susceptibility to infection as a side effect. Therefore, in this study, dexamethasone is not used for acute myeloid leukemia and myelodysplastic syndromes as an antiemetic agent. None of 100 patients in this study were treated with dexamethasone as supportive therapy. In clinical practice, 5-HT_3_RAs and metoclopramide are the preferred antiemetic agents in azacitidine-containing regimens.

The results of this study showed that the 5-HT_3_RA group had significantly higher rates of TC, CC, and CR (Table 2) and exhibited a significantly delayed time to first emetic episode in comparison with the metoclopramide group throughout the course of azacitidine monotherapy and that of azacitidine combined with venetoclax therapy (Fig. 1). Thus, in azacitidine and azacitidine plus venetoclax therapies, the use of 5-HT_3_RAs may exhibit a greater antiemetic effect than that of metoclopramide. Nausea and vomiting caused by chemotherapy, such as azacitidine treatment, is thought to be attributed to the binding of serotonin to 5-HT₃ receptor distributed mainly in the upper gastrointestinal tract. Serotonin is released from the gastrointestinal mucosa stimulated by anticancer drugs. Anticancer drugs also directly stimulate the chemoreceptor trigger zone located at the bottom of the fourth ventricle and activate the vomiting center in the medulla oblongata via dopamine D₂ receptor and NK1 receptor [11]. It has been reported that 5-HT₃RAs exhibit superior antiemetic effects compared with metoclopramide in cisplatin-based therapies [12].

In our study, vomiting occurred in 2 patients in the 5-HT_3_RA group and 4 patients in the metoclopramide group. On the other hand, nausea was reported in 4 patients in the 5-HT_3_RA group and 14 patients in the metoclopramide group, and all cases were Grade 1, resulting in similar rates between CC and CR (Table 2). Metoclopramide acts as both a dopamine D_2_ receptor antagonist and a 5-HT_3_RA [13]. Although 5-HT_3_ receptor antagonism of metoclopramide has been suggested at very high doses (≥ 2 mg/kg) [14], no reports indicate this effect at a dose of 5 mg/day, suggesting that 5-HT_3_ receptor antagonism is not a primary mechanism of the antiemetic action of metoclopramide at this dose level. In the study by Uchida et al. evaluating the efficacy, safety, and pharmacokinetics of azacitidine in Japanese patients, nausea has been reported to occur in 34.0% (18/53) of patients despite the use of 5-HT_3_RAs [5]. In the present study, the incidence of vomiting was 6.25% (2/32) in the 5-HT_3_RA group, which was less than that in previous clinical trials [5]. It is crucial to inform patients beforehand that antiemetic therapy for anticipatory nausea and vomiting will be implemented before starting chemotherapy [3]. In clinical practice, patients are informed about the potential adverse effects of azacitidine, including nausea and vomiting, and about the recommended antiemetic therapy, based on findings from clinical trials involving Japanese patients. This approach may help alleviate their anxiety through a better understanding of the treatment. In addition, young age, female sex, non-habitual alcohol intake, a history of motion sickness, and morning sickness in females have been identified as patient-related factors affecting the effectiveness of antiemetic therapy [15, 16]. In this study, detailed data on patient-related factors other than age and sex are lacking. In this study, patients in the 5-HT_3_RA group were administered with either ramosetron (0.1 mg) or granisetron (3 mg). Previous studies have suggested that ramosetron (0.1 mg) has an antiemetic effect comparable to granisetron (3 mg) [17]. In the present study, we evaluated both patients receiving azacitidine monotherapy and azacitidine plus venetoclax regimen. The emetic risk of venetoclax is mild [3], suggesting that the concomitant administration of venetoclax may not largely affect the incidence of vomiting or nausea.

In addition, azacitidine in this study was administered via either subcutaneous or intravenous infusion. It has been reported that there is no difference in the incidence of nausea caused by azacitidine between subcutaneous and intravenous infusion [5]. Side effects of 5-HT_3_RA include constipation, headache, dizziness [18], and QT prolongation [19], whereas those of metoclopramide include tardive dyskinesia and QT prolongation [9]. However, evaluations of these side effects were difficult, because many patients also used laxatives and hypnotics.

Limitations of this study include the retrospective analysis, the single-center design, and the limited sample size, which may limit the generalizability and lead to bias between the two groups. Age, sex, alcohol consumption, history of motion sickness, and history of morning sickness are known to be risk factors for CINV [15, 16]. As shown in Table 1, age and sex were not significantly different between two groups. However, alcohol consumption, history of motion sickness, and history of morning sickness could not be assessed, because such information was unavailable in the electronic medical records. In this study, the results were not adjusted for these confounding factors. Therefore, further investigation in large prospective studies is needed.

In conclusion, in chemotherapy with azacitidine, 5-HT_3_RAs are suggested to have better antiemetic effects than metoclopramide.

## Data Availability

The datasets used during the current study are available from the corresponding author on reasonable request.

## Abbreviations

CC: complete control
CINV: Chemotherapy-induced nausea and vomiting
CR: complete response
JSCO: Japan Society of Clinical Oncology
NCCN: National Comprehensive Cancer Network
NK1: neurokinin-1
TC: total control
5-HT_3_RA: 5-hydroxytryptamine 3 receptor antagonists
